# The relationship between dominant Western discourse and personal narratives of female genital cutting: exploring storytelling among Swedish-Somali girls and women

**DOI:** 10.3389/fsoc.2023.1188097

**Published:** 2023-07-11

**Authors:** Camilla Palm, Eva Elmerstig, Charlotta Holmström, Birgitta Essén

**Affiliations:** ^1^Centre for Sexology and Sexuality Studies, Department of Social Work, Malmö University, Malmö, Sweden; ^2^Department of Women's and Children's Health (IMHm), Uppsala University, Uppsala, Sweden

**Keywords:** female genital cutting, female genital mutilation, anti-FGM discourse, migration, storytelling, qualitative research

## Abstract

**Introduction:**

A dominant narrative, referred to as “the standard tale,” prevails in popular representations about female genital cutting (FGC) that often contrast with how cut women traditionally narrate their FGC experience as meaningful in contexts where FGC is customary. However, scholarship has increasingly highlighted how global eradication campaigns and migration to countries where FGC is stigmatized provide women with new frames of understanding which may lead to a reformulation of previous experiences. This article subjects the storytelling itself to analysis and explores how participants narrate and make sense of their FGC experience in a post-migration setting where FGC is stigmatized.

**Methods:**

Semi-structured focus groups (9) and individual interviews (12) with Swedish-Somali girls and women (53) were conducted.

**Results:**

The article highlights how the participants navigate their storying in relation to "the standard tale" of FGC in their efforts to make sense of their experiences. Navigation was conducted both at an intrapersonal level through continuous identity work, and in relation to the social context in interpersonal encounters, i.e., with service providers and others, among whom the standard tale has become a truth.

**Discussion:**

The article places the analysis within broader discussions about anti-FGC work and considers the implications in relation to efforts to end FGC.

## 1. Introduction

Through decades of global efforts aimed to counter female genital cutting (FGC), a uniform portrayal of the practices has taken form within an international discourse of opposition that frames FGC as gender-based violence that victimizes girls and women due to its presumed negative consequences to women's wellbeing (Leonard, 2000; Shweder, 2005). This common framing of FGC, however, contrasts with how cut women traditionally narrate their experiences as meaningful according to concepts of proper identity related to gender, ethnicity, culture, and/or religion in regions where FGC is customary (Abusharaf, 2001; Njambi, 2004, 2007; Jirovsky, 2014; Powell et al., 2020). While anti-FGC messages—through global eradication campaigns and migration processes—have contributed to promoting negative attitudes toward FGC among once-practicing groups (Hodžić, 2017; Wahlberg et al., 2017; Powell and Yussuf, 2018), studies have increasingly highlighted that exposure to adverse messages about FGC may negatively impact girls' and women's self-understanding (see review by O'Neill and Pallitto, 2021). Drawing on these recent concerns, this study explores how girls and women of Somali background narrate their FGC experience post-migration to Sweden—a country with a condemnatory discourse on FGC. More specifically, the study highlights how interviewees relate to dominant Western discourses and related representations about FGC when storying and trying to make sense of their own FGC experience.

In the article, “discourse” is used to denote knowledge, i.e., a particular set of ideas, assumptions, conceptualizations, and framings of a phenomenon that are collectively shared and taken-for-granted as truths (Foucault, 1981). The terms “narrative,” “story,” or “account” are used interchangeably to refer to explicit ways of framing experiences or a phenomenon in terms of lengthy textual or speech representations such as interviewees' accounts or media reporting. This article will engage in a particular narrative that has variously been referred to as “the standard tale” of FGC (Leonard, 2000, 2020), “standard story” (Njambi, 2007), or “FGM fantasy” (Rogers, 2009). Although not identical in their meanings, the concepts all refer to popular representations and truth claims connected to dominant condemnatory discourse on FGC.

## 2. Narratives about female genital cutting

### 2.1. A dominant Western narrative about FGC: “the standard tale”

“FGC” (or female genital mutilation or female circumcision) is an overarching term for a variety of genital alteration procedures performed on girls for non-medical purposes. It is practiced among different groups, for different reasons, and under varied circumstances (Box 1). Despite its diversity, FGC has come to be portrayed in a now familiar way, commonplace in anti-FGC work, policy papers, media reporting, and introductory sections in FGC literature (Leonard, 2000; Gruenbaum, 2005; Njambi, 2007; Rogers, 2009; Wade, 2009; PPAN, 2012; Malmström, 2016). The American sociologist Lori Leonard has argued that this narrative has become so standardized in its form, she has termed it “the standard tale” of FGC (Leonard, 2000, 2020). In her article, “We did it for pleasure only”: hearing alternative tales of female circumcision', Leonard identifies some common features of the standard tale:

What is cut, how much is cut, at what age it's cut, with what implement, and by whom […] So is the litany of consequences: hemorrhage, shock, infection, infertility, sexual dysfunction, problems in childbirth, death, and so on. (Leonard, 2000; p. 213)

Box 1 **WHO typology of “female genital mutilation” and Somali categorizations and terminology.**

‘Female genital cutting' refers to a broad range of procedures which have been practiced in varied ways, for varied reasons, under different circumstances among some groups within some African, Asian and Arab countries (such as Sudan, Somalia, the Gambia, Egypt, Malaysia, Senegal, Kenya, Eritrea, and Ethiopia). Practices are diverse but are generally divided into four categories according to the World Health Organization typology (WHO, 2018): *infibulation* where the labias are trimmed and sutured together with or without cutting involving the clitoral gland (type III cutting); *excision* of the labia minora/majora with/without cutting parts of the clitoris (type II), or excision of the clitoral hood and/or the tip of the clitoris (type I). Type IV includes all other procedures that injures the outer genitalia for non-medical purposes such as pricking (inducing a drop of blood with a sharp object on the clitoral gland), piercing, incising, cauterization, burning (WHO, 2018). In Somalia, FGC [*gudnin* in Somali] has traditionally been categorized into two broader types: infibulation (or pharaonic cutting) or sunna cutting, that may include all other forms of cuttings other than type III. In practice, sunna cutting may entail an essential closure of the vaginal orifice, anatomically equal to pharaonic cutting (WHO, 2018).

In the standard tale, the practices are typically described as ancient and deeply rooted (Leonard, 2000, 2020; Njambi, 2007). Based on an understanding of FGC, more often referred to as “female genital mutilation” (FGM), as a manifestation of men's domination over women, typically, cut women are narrated as victims of gender-based violence. Parts of the narrative are epitomized in the Somali supermodel Waris Dirie's bestselling novel *Desert Flower* (Dirie and Miller, 1998):

The operations are usually performed in primitive circumstances by a midwife or village woman. They use no anesthetic. They'll cut the girl using whatever instruments they can lay their hands on: razor blades, knives, scissors, broken glass, sharp stones—and in some regions—their teeth. The process ranges in severity by geographic location and cultural practice. The most minimal damage is cutting away the hood of the clitoris, which will prohibit the girl from enjoying sex for the rest of her life. (Dirie and Miller, 1998; p. 218)

In Njambi's (2004) article targeting the anti-FGC campaign and the discourse it has produced, she poses a critique of what she terms “sensational stories” about FGC. As regards a publication from the American Medical Association (AMA) in 1995 where teeth are mentioned, Njambi critically notes “while not a single group is identified which employs such crude instruments, such statements are presented as a matter of ‘fact' from one text to another in almost identical language” (Njambi, 2004; p. 284). Images of unsanitary conditions, rusty razor blades, coercion, and violence from the ones you love, such as in Dirie's story, are common features in FGC representations (Gruenbaum, 2005; Rogers, 2009). So are medical and sexual health consequences (PPAN, 2012). It is notable, however, that the question about health outcomes related to FGC is not as intuitive as it might seem from the “standard tale” (see, e.g., Essén, 2023). Systematic reviews on health outcomes show that FGC is associated with some health consequences, with type III cutting being associated with more health and sexual complications than type I and II cuttings (Berg et al., 2014; Lurie et al., 2020; Sylla et al., 2020; Johnson-Agbakwu et al., 2023). Nevertheless, for many health conditions, no statistically significant associations can be found (Berg et al., 2014; Lurie et al., 2020; Sylla et al., 2020). This is probably not a result of all FGC being free of harm but reflects the challenges in conducting methodologically and conceptually sound research on the matter (e.g., representative sampling, adequate control group, accounting for the diversity of FGC practices, using tools that account for local meanings of FGC and capture the complex interplay among culture, health, and sexuality). Despite these reservations regarding existing studies, assertions about long-term health consequences are often presented as “facts” in the standard tale (Essén and Mosselmans, 2021; Essén, 2023).

This way of describing FGC is part of a larger discourse of opposition toward FGC that has its legacy in early Western radical feminist writings about the practices, sometimes referred to as the “anti-FGM discourse” (Njambi, 2001). FGC became an issue of global interest when radical feminists started in the 1970's portraying the practices to rally support for its abandonment. Particularly important was the work of Fran Hosken who coined the term “female genital mutilation” (Hosken, 1993). Theoretically anchored in patriarchal theory, first-hand accounts of FGC were analyzed as a manifestation of men's oppression over women, often emphasizing the potential harms of the practices, especially targeting sexual health. Within this framework, practices were seen as inherently abusive and something women cannot truly consent to (Thiam, 1978; Daly, 1979; Hosken, 1993). Personal narratives that challenged these ideas were dismissed as inauthentic (e.g., Thiam, 1978; Hosken, 1993), and female proponents of the practices were described as “mentally castrated” (Daly, 1979; p. 164). In her discourse analysis on feminist contributions to the FGC issue, the American sociologist Lisa Wade notes that the work of Hosken and her contemporaries has largely come to shape current international efforts to end FGC, and likewise the public debate of today (Wade, 2009, 2012, see also Earp and Johnsdotter, 2021). In the Swedish context, the anti-FGM discourse is hegemonic. The perspectives in this dominant discourse have been influential in informing political policy and law (SFS, 1982; The Swedish Government, 2016; Ministry of Health and Social Affairs, 2018; p. 316) and the representations of the standard tale—as a manifestation of this discourse—prevail in Swedish educational materials on FGC, official reports, and statements by Swedish authorities (e.g., National Board of Health and Welfare, 2002; National Board of Health Welfare, 2005; Östergötland County Administrative Board, 2015)^1^. The standard narrative has also influenced societal responses, public awareness, and professional practice, insofar as professionals expect the standard tale from affected groups during professional encounters (Johnsdotter, 2019; Palm et al., 2019, 2020, 2021).

### 2.2. Personal narratives about FGC experiences in research

While the “standard tale” has informed popular beliefs about FGC and proven effective to mobilize action for change and political awareness (Gruenbaum, 2005), such accounts are by necessity simplistic in their nature—not aimed to capture nuances or diversity of voices (Obiora, 1997; Nnaemeka, 2001; Gruenbaum, 2005). Rather, as “formula stories,” they provide an image stripped of much of its context (Loseke, 2011). Meanwhile, empirical accounts among women in social science research have offered accounts displaying diverse meanings and contextual understandings of the practices, including variations in cutting procedures, motives, and functions among the varied groups that practice it or have once practiced it (Box 1). For example, some women may say that reasons for practicing FGC include beautification and modernization (Leonard, 2000, 2020), or that it is about health and fertility (Ahmadu, 2007; Jirovsky, 2014). Other women might emphasize sexual morals or the importance of FGC in developing a proper gender identity, religious identity, or cultural or national identity (Abusharaf, 2001; Njambi, 2004, 2007; Malmström, 2016; Powell et al., 2020), or a combination of the above. There are also women in FGC-practicing contexts—activists and others—who long have discarded the alleged benefits of the practices and strived for its abandonment (Abusharaf, 2000). Moreover, how women relate to their experience of FGC may vary greatly, from feelings of strength, pride, honor, and empowerment, to feelings of sorrow, grief, betrayal, and regret (Abdulcadir, 2019). On an individual level, various factors may influence how one makes sense of the FGC experience. Such factors may include the presence of long-term health consequences or other health conditions (Catania et al., 2007; Abdulcadir, 2019; Essén and Mosselmans, 2021), how well the girl was prepared for the event, degree of cultural acceptance in the context it was performed (Schultz and Lien, 2013, 2014), and use of anesthesia and other circumstances around the cutting event (Abdulcadir, 2019). In other words, research shows a great variation in how women have narrated their FGC experience: as victims of violence, coercion, and betrayal, or in terms of empowerment, enhancement, and female emancipation. What stories are narrated in a particular context depends both on individual circumstances and biographies and on structural and sociocultural conditions shaping experiences (Plummer, 1995; Gubrium and Holstein, 2009).

### 2.3. Reformulation of experiences in migration

While research thus has documented endorsing accounts of cutting practices in settings where FGC is a social norm, studies suggest that a migration context with a condemning discourse on FGC offers a powerful potential site for the renegotiation of previous values. Several studies now exist that have investigated affected groups' attitudes toward FGC in Western migratory settings, reporting processes of abandonment (e.g., Gele et al., 2012; Koukoui et al., 2017; Wahlberg et al., 2017; Johansen, 2022). A related question that has gained increased attention among scholars is to what extent normative discourses on FGC and anti-FGC politics also can have a negative impact on girls' and women's self-understanding (Ahmadu, 2007; PPAN, 2012; Johnsdotter and Essén, 2016; O'Neill and Pallitto, 2021). Research in migratory settings where FGC is stigmatized indicates that women may internalize new understandings of their experiences. For example, in studies about sexuality, cut women have reported how exposure to new cultural scripts on FGC and body appearance had made them start questioning their normalcy, leaving them feeling bad about themselves (Johnsdotter and Essén, 2004; Ahmadu, 2007; Catania et al., 2007; Lien and Schultz, 2013; Villani, 2017). Through global eradication campaigns, similar tendencies have been reported among women in Ghana, Egypt, and Burkina Faso (Jirovsky, 2014; Malmström, 2016; Hodžić, 2017).

While previous research has suggested that exposure to anti-FGC discourse may result in women internalizing an understanding of themselves as maimed, this study contributes to further understanding of girls' and women's agency in constructing and telling stories about FGC in a social setting with a stigmatizing discourse on FGC.

## 3. Method

Nine focus group discussions (FGDs) with 2–17 participants each and 12 individual interviews with Swedish-Somali girls and women (16–65 years old) were conducted for a total of 53 participants. Most participants interviewed individually had also participated in an FGD. Some had lived in Sweden for many years whereas some had migrated to Sweden more recently. Most of the younger women attended or had recently completed upper secondary school. Some had full or part-time jobs or were unemployed, and a few studied at the University. Among the older women, a few had full-time employment and some had part-time employment, while others were unemployed or were enrolled in educational programs. The composition of participants reflects the situation in Sweden, where older Somali women have faced barriers entering the Swedish labor market (Carlson et al., 2012). In the article, participants between 16 and 25 years of age, often but not always unmarried, will roughly be referred to as “young women.” Other participants will be referred to as “older women.” While most of the older participants reported they had pharaonic cutting (type III), generally, younger participants had sunna cuttings or were uncut.

Participants were recruited from across the country. To include people with a variety of experiences, participants were approached through different vantage points. Most were invited to participate through the help of female representatives at three organizations frequented by Somali women, of which one was a youth organization specifically working with FGC (i.e., awareness-raising and support for girls with FGC). The representatives advertised the project, approaching members of the organizations, friends, acquaintances, neighbors, and other women they knew. Younger participants were also invited through school nurses working with young migrants and through a specially trained female Swedish-Somali research assistant using her network from her work as a Somali health-communicator in Sweden. Yet, others were recruited through previous contacts.

Interviews were semi-structured with open-ended questions covering queries about previous knowledge and information about FGC, views on how FGC relates to health and sexuality, and the benefits and disadvantages of the practices. All interviews were conducted face-to-face by the first author and were conducted at facilities used by Somali community organizations, schools, libraries, or at participants' workplaces, as preferred by the interviewees. Interviews were held in Swedish, English, and Somali. In FGDs, discussions in Somali were immediately summarized into Swedish by the Somali-speaking women helping with recruitment and cross-checked with other Swedish-speaking participants. At one point, a professional interpreter was used. Two individual interviews were held mainly in Somali and were interpreted by an acquaintance chosen by the interviewee. Individual interviews lasted on average 60 min and FGDs lasted 1.5–3 h. Often but not always groups were held with young (often unmarried) women and adult women separately. With consent from participants, interviews were audio-recorded. In some cases, notes were taken, and interviews were summarized immediately after the interview.

The current study is part of a PhD project that initially focused on young women (Ethical clearance Dnr 2014/620). The project was gradually extended to also include older women. Ethical approval was provided by the Swedish Ethical Review Authority (Dnr 2020-00724). The present article is based on interviews conducted in two sets between 2014 and 2022, with the bulk of data being collected in 2020–2022. Participants gave verbal informed consent at the beginning of the interview, which was documented by audio recording. Participants were provided with an information sheet about the purpose and procedure of the study which was read together and discussed before the interview. The information included the right to withdraw the participation at any time without further explanation, information that participation is voluntary, and that participants may choose not to answer questions, would they wish so. All interviewees have been given pseudonyms and details have sometimes been changed in the presentation of the findings to maintain confidentiality of the participants. For a few participants, more than one pseudonym was assigned.

The study approached the analysis assuming that stories are co-produced and socially constructed (Plummer, 1995; Gubrium and Holstein, 2009). Departing from this assumption, analytical attention was paid both to *what* people say, and to *how* people say it, i.e., the meanings conveyed by the strategies employed when constructing stories (Gubrium and Holstein, 2009), and also to the interactional processes involved in the telling (Plummer, 1995). Given that FGC is banned, morally condemned, and a stigmatized experience in Sweden, participants could be expected to assume that the interviewer holds expectations on a particular story about FGC to be brought forward during interviews. Acknowledging that the interview situation is affected both by the social context and the teller's expectations of the listener (Plummer, 1995), careful efforts were made to make room for a variety of experiences to be voiced. For instance, questions were posed in a way meant to signal space for various stories, such as “I know that some.. while others… What do you think?” Nevertheless, the issue of self-representation is always present in research. As will be shown and discussed in the Results section, stories are always created, negotiated, and exchanged in relation to the social context and to the receiver of the story (Linde, 1993; Plummer, 1995).

Guided by these approaches to storytelling, an inductive analysis was conducted. Transcripts were read and coded, checking for recurring themes related to the study topic. In the first step of the analysis, the attention moved back and forth between the *whats* and the *hows*, and refining codes and themes, what has been called “analytic bracketing” (Gubrium and Holstein, 2009). The Results section was structured through the themes identified during this process of analysis. Themes were thereafter refined during the process of writing, developing subsequent themes and sub-themes. In the second step of the analysis, the focus was on how the accounts related to dominant Western discourses on FGC, including the “standard tale” of FGC. To analyze how dominant discourses were related to the interviewees, bell hooks' (1989) concept of *talking through* and *talking back* was used. It suggests that dominant discourses may be used (talked through) or refused and resisted (talked back to) and may help illuminate the constructive elements people use when making sense of and constructing stories of their life experiences.

## 4. Results

The analysis is structured under the following themes addressing how participants navigated dominant Western discourses on FGC: (1) Positioning own experiences to an image of the infibulated woman; (2) Narrating the FGC event as a commonplace story; and (3) Stigma.

### 4.1. Positioning own experiences to an image of the infibulated woman

When talking about FGC practices, participants of all ages commonly used the Somali terms *gudnin*, and “sunna” and “pharaonic,” respectively, to denote the various practices included under the overarching term “FGC.” Few participants used “FGM,” “mutilation,” or the Swedish equivalent [*könsstympning*]. It did occur among those who had been engaged in anti-FGC work. When *könsstympning* was used, it was described as “the Swedish term.” To reflect the tone of the interviewee, the terms used by the individual interviewee will be used in the presentation of the empirical data, e.g., “sunna,” “sunni,” “pharaonic,” “mutilation,” “circumcision,” etc.

A salient theme in the interviews was that participants negotiated notions of FGC as harmful to health and sexuality. Framed within a medical and religious narrative, interviewees condemned FGC, referring to it as “dangerous,” “traditional,” and “cultural” (as opposed to “modern” and “religious”). Many cited health risks associated with FGC, asserting, for example, that it “comes with a lot of problems,” “causes ‘pain',” and a “lack of sexual feeling.” Their position was attributed to a conviction that it is a harmful and unreligious practice, not least in relation to Islamic notions of “doing no harm.” Participants explained how notions of harm were based on personal experiences—their own, or heard from significant others, or through circulating stories about women with FGC in their local context, when in Somalia and/or in Sweden. Sometimes, these stories were framed in the form of “horror stories” of death, “lifelong suffering,” severe complications, painful wedding nights, “sexual death,” and broken marriages due to problems associated with FGC. The participants' position was also informed by anti-FGC discourses in Sweden, e.g., through campaigns, targeted sessions about FGC in schools, or anti-FGC initiatives targeting the Somali community, or more seldom through news media. Maano (18–23 years, unmarried) said in an FGD:

When I came to Sweden, I was 14, I was invited to a meeting after class. They talked about the law, that it is forbidden, of everything that can happen and the risks and everything—then I was informed about it. Because I knew nothing about it [before].

Similarly, Aalihya (18–23 years, unmarried) explained in an individual interview:

Interviewer: What kind of information do you receive about this in school? What have you learned about gudnin [FGC] in Sweden?

“Before I came here, I didn't think that this would affect my life in any way. I was 13 years old and hadn't heard anything about gudnin, because it was something no one talked about … So when I came here, I learned quite a lot: that you can have difficulties during sex … that it affects our ability to feel pleasure during sex, that it's not endorsed by the religion, which has been a major reason for FGC.”

In addition, interviewees reported how they were influenced by discursive changes in the Somali group in the diaspora and in Somalia in which pharaonic cutting increasingly has been condemned, e.g., by imams, or in campaigns. Based on these sources, an image of the “cut woman” was constructed. In terms of harm, personal experiences of FGC were positioned against and made sense of in relation to this imagined other (infibulated) woman.

Implicit in these narratives was that “FGC,” if not specified, generally was associated with pharaonic cutting. This was made visible, for example, in how health consequences were discussed as an obstruction due to a vaginal closure characteristic of pharaonic cutting. Common comments were that sunna cutting was “less harmful,” “nothing,” or “just a nick.” The exception was some younger uncut participants who tended to collapse all genital cutting under the umbrella “FGC,” or others who explicitly included sunna cutting, often in the context of stating opposition against all forms of genital cutting. In terms of their own experiences, discourses on health and sexuality were negotiated in relation to personal experiences of not necessarily feeling harmed themselves. When participants narrated their experiences, the presence of a seal was particularly central and personal experience was often related to how they imagined it to be for (other) infibulated women. The positioning varied primarily depending on the participant's own experiences of FGC.

Young women with sunna cutting made sense of their experiences of FGC by positioning it against pharaonic cutting (and not, for example, uncut women). Sometimes the positioning was explicit. This was most evident in Yasmiin's narrative. Yasmiin (18–23 years, unmarried) was interviewed together with a friend from a non-practicing ethnic group. During the interview, she was careful to distance her experience of sunna cutting from pharaonic cutting and what she assumed were societal expectations of FGC. She described pharaonic cutting as something causing menstrual pain and sexual problems and simultaneously dissociated from an idea of herself as having been harmed—framing her sunna cutting as a harmless form of FGC:

Yasmiin: well my [FGC], it's much better, or you know, it's not sewn all together. It's just like with the boys.' They just cut a bit and then they didn't touch anything.

Friend: But do you feel anything (sexually)?

Yasmiin: Yes, of course! You know, I'm like anyone else. Only that they touched a bit, but they didn't sew.

With statements such as “this sunni, they just cut a tiny bit, so you mustn't feel sorry for me,” and “I don't think it can be compared with those who are sewn,” she distinctly “talked back” to discourses about cut women, stressing that it is not a monolithic experience. She further refused a categorization of herself as “other,” instead emphasizing sameness and similarities between her and uncut women. She said, “I don't feel any different, I don't feel different down there.” By these remarks, Yasmiin resisted the idea of herself as victimized, harmed, or sexually maimed.

While not all young or older women with sunna cutting explicitly positioned their experiences against pharaonic cutting in this manner, the positioning was often made indirectly through relating their own experiences to that of an imagined, other infibulated woman. Most young and older women with sunna cutting dis-identified with “FGC,” sensing that the topic was not related to their own experiences. For example, Aalihya (18–23 years old, unmarried) who first canceled the interview, explained:

At first, I thought of not coming so I canceled [the interview], because I thought I might not have anything to contribute. As I'm not pharaonic, I cannot speak [for women with FGC] and don't want to speak for someone else who has real problems.

While many young women voiced how “FGC must end,” cataloging potential health problems, most participants simultaneously discounted their own experiences of sunna cutting as a form of FGC that had caused them long-term harm. Instead, what they had learned about the long-term health effects of FGC was spontaneously related to pharaonic cutting, and in this way also seen as unrelated to their own experiences of sunna cutting. Hani (16–18 years, unmarried) expressed it thus:

[With gudnin] there are so many bad things that can happen to your body. It can impact your body, it can affect your health, it can affect how you pee, it can affect your period, everything… and I think the worst thing is the first night when you get married, that's the worst thing.

Interviewer: Are you yourself worried about this?

Hani: Me? No, I'm not worried, but I'm thinking about how it is for the others.

Many of the young interviewees used the term “them” as opposed to an implicit “us” or “me,” when they narrated what FGC meant to them. Common comments were about feeling “fortunate” or “being grateful” that their parents had avoided pharaonic cutting and chosen sunna cutting for them instead. In contrast, pharaonic cutting was described with pity and sorrow. Young women expressed worry and sympathy for mothers, friends, and others with infibulation. Some imagined that infibulation would invoke shame or be a too-sensitive topic to discuss for infibulated women, as they imagined it to affect women's everyday lives, creating a “life-long suffering” or a “sexual death,” as described by one interviewee. In these examples, the young women with sunna cutting *talked through* dominant discourses of FGC as a harmful practice in the long term. The way participants related it exclusively to pharaonic cutting while excluding sunna cutting suggests that they were influenced by the discursive change from pharaonic to sunna cutting in Somalia, more so than by Western dominant discourse that also tends to emphasize harm imposed by interventions on the clitoris. Through this construction, it could be interpreted that the stigma of anti-FGC discourse was placed elsewhere—onto pharaonic cutting, which provided a yardstick against which their own experiences were storied. Yet, others expressed reluctance to divide experiences into sunna or pharaonic cutting, claiming the practices are the same in terms of anatomical change, stating that some so-called sunna cuttings still involve partial or essential closure of the vaginal orifice. This was even more so among the older women with sunna cutting, who often stated that there were only small differences between their sunna cutting and pharaonic by the time they were cut.

Few of the younger participants with sunna cutting thus narrated their experiences in terms of feeling harmed. More common was expressing doubts and worries about whether the FGC might affect them in future. These concerns were related to marriage and child-birth, and often related to being partially sewn closed. Yet, a few also worried about how sunna cutting would affect sexual desire and pleasure. In terms of health and wellbeing, when young participants with sunna cutting positioned their experience against pharaonic cutting, sunna cutting was simultaneously constructed as undramatic or unproblematic. The narration of one's own sunna experience as something that had not affected their health and wellbeing in any essential way did not, however, mean that participants necessarily stated support for its continuation. With only one exception, young participants with sunna cutting stated opposition against all forms of FGC, but not based on notions of long-term harm. Instead, opposition toward sunna cutting was expressed as the procedure being a painful ordeal in the short-term that no longer had any tangible meaning, as social pressure had eased with migration and religious teachings had made participants reassess the practice on a religious basis.

#### 4.1.1. “It gets better after the first child”

The presence of a seal was central also in the narratives of infibulated girls and women. Many mentioned health complications they associated with being partially or essentially sewn closed, e.g., genital pain, pain and difficulties during menstruation and first sexual intercourse, childbirth, and/or urination. Others mentioned associated impacts on sexual desire and pleasure, relating to a socially ideal gendered norm of women with infibulation as sexually modest and passive, although many refuted this idea, stating other experiences. Once the seal was opened, however, many stated “everything will be alright.” Fawsia (40–45 years, married) who reported having been partially sewn closed during “sunna” cutting, said: “After I married, it's all good now. Now it's ok.” Anissa (16–18 years, unmarried) who associated infibulation with difficulties during sexual intercourse and childbirth, looked forward to her planned defibulation surgery. She stated:

You will be in pain [during sex] and have no sexual feelings. But they'll [the feelings] come back if you open [the infibulation]. I believe that.

Interviewer: So as long as you open [the infibulation], then there's no problem?

Anissa: Yes.

Anissa echoed narratives among the older women with pharaonic cutting, many of whom expressed great concern over having been sewn closed; however, making remarks that difficulties were resolved after defibulation.

While many described experiences of harm, others dissociated from such experiences. In addition to these narratives, personal experiences were related to the presence of a seal (and not, for example, the excision of labia or the clitoral gland). Farhiya (30–35 years, married), for example, made sense of her absence of pain or troubles by narrating it as an effect probably due to the fact that some stitches had opened up after her surgery as she was out playing and running after the operation. Refuting the idea of herself as harmed, she still talked through Swedish and Somali narratives that associate infibulation with inevitable long-term harm.

In terms of health problems, the meanings of FGC were contested among participants. Sometimes, they claimed ordeals of infibulation to be a collectively shared experience, Asha (50–55 years, married) expressed it thus in an FGD: “we're all in the same boat, we have been through this circumcision,”' suggesting she expected others to share similar experiences. In this sense, Asha talked through a dominant, supposedly known narrative. At other times, women such as Zahra (40–45 years, married) resisted generalizations, expressing in-group variations and FGC as a heterogenous experience. This was evident in FGDs among older women when some participants claimed to speak the “truth” about FGC—either it was stated as unavoidably carrying health problems or people emphasized FGC as a heterogenous experience:

Woman A: It hurts very much the first time you have sex.

Woman B: But it doesn't continue after that, it's after a few months, [changes her mind] only the first time, then it disappears, that pain.

Woman C: We're all different, some are still in pain you know, others are not. So there you are different as individuals, you know. We're not all the same.

Woman D: If you're healthy, some are healthy, they don't feel any pain. [Interpreter summarizes:] “That woman says that she is still in pain from time to time. ‘Sometime it's there, sometimes it's gone.' But it is different for different women.”

Yet, others compared their experiences with uncut women, pondering about genital pain, menstrual pain, or sexual arousal among those who have not undergone FGC:

Woman, older: We're all in the same boat, we've been through circumcision, but we want to know what it's like for women without circumcision.

This group of girls and women expressed uncertainty about whether to interpret the presence of discomfort to FGC or not.

### 4.2. Narrating the FGC event as a commonplace story

Many participants, young and older, told stories about the moment of the cutting event in their own lives. When narrating their experiences, participants negotiated their personal experiences and also navigated storytelling in relation to assumptions about the listener's expectations and to dominant Western narratives about FGC and FGC experiences. In interviews, the FGC event was recurrently described as an expected and ordinary part of life, what can be called a commonplace story. For example, Jawahiir (45–50 years old, married) expressed it thus:

When I had this gudnin, when I was a kid, that was ok for me to have gudnin because it's religion and all the other girls also had it.

Although many, such as Jawahiir, expressed that FGC was a painful ordeal, emphasis in the accounts was, with only a few exceptions, placed on the naturalization and anticipated-ness of the event. When participants were asked about their previous knowledge of FGC, a common comment was that FGC was something “everyone went through,” conveying a sense that life was expected to go on. Manoo (18–23 years, unmarried) commented:

Before I came to Sweden, this was something I never thought about. It was just something everyone had done. I didn't think that it would affect my body in any kind of way, or affect anything, it was just something that happened to all [girls].

Through contextualization, participants rationalized their position by sharing an understanding of the cultural context in which FGC was a social norm. About the radically different discourses on FGC in Somalia and in Sweden, Aalihya (18–23 years, unmarried) said:

I don't feel like I'm proud that I have [FGC] but I think that this was their way, where I lived. And people are like that, that they can't do everything right all the time, so I can't say that I'm proud, or that I have had something that is beautiful or anything. That's not how I think. But at the same time, I think its like, I'm not the person who thinks that “Oh my God, I have been through some horrible stuff!” I accept [it] because I think that they didn't know any better.

Through unprompted statements such as that of Aalihya or Maano, it seemed like participants commented on an expected grander narrative about how girls and women would relate to their FGC in childhood. In constructing the stories in this way, participants seem to speak to and negotiate what they assume to be shared ideas of FGC as the opposite (e.g., as a disruptive, violent, and malicious event)—prompting them to make the clarifying remark implicitly stating, “it wasn't like that.”

Many participants engaged in similar talking back to imaginations of FGC as unavoidably associated with feelings of victimization. Others, such as Khadra (50–55 years old, married), were one of the few who clearly emphasized a victimized perspective. She participated in interviews on more than one occasion. During the first individual interview she was crying, saying “What I was exposed to was indeed very terrible, they took away everything.” Khadra stated that memories keep coming back. Throughout the interview, she emphasized the harm and sorrow she felt over FGC, such as health and sexual problems, and the recollection of the loss of close ones after FGC.

Another recurrent theme stressed was how participants had looked forward to the day of FGC, given the context. Many emphasized their self-determination and desire in relation to having FGC performed. Haaweyo (16–18 years old, unmarried) remarked:

You know, it was *we* [sisters] who asked [our mom], she didn't force us to do this. We were the ones who thought we should do this, because everyone in the neighborhood would bully us if we hadn't done it.

Many described the moment of FGC as a painful event they regret, yet like in Haaweyo's account, the FGC event was primarily made sense of by stressing that it was something they felt happy about, anticipated, begged for, and were looking forward to, challenging the idea of themselves as a passive victim of violence.

While many participants underlined their agency in relation to the cutting event, they simultaneously made sense of it as something out of their control, something “overdetermined,” that they had tried to resist in various kinds of ways. A desire to be like everyone else coupled with peer pressure and fear of stigmatization were some elements brought forward when participants described their desire to undergo FGC. Mothers who opposed having their daughters go through cutting also often explained how grandmothers decided on FGC against their will while living in Somalia. Jawahiir (45–50, married) explained:

When I had this gudnin, when I was a kid, that was ok for me to have gudnin because it's religion and all the other girls also had it. But when I grew up and got married, I thought this was wrong. That you shouldn't cut. So when I had my first daughter and my mother told me I must have her cut, I told her you shouldn't cut. But she said “no, it's religion, it's important,” so I'm like “ok”.

Through their storytelling, many participants refused victim status and emphasized agency, yet also described the position in relation to structural conditions such as cross-generational power relations and social pressures that constrained their power to prevent the event from happening. Countering ideas of themselves as passive victims, participants constructed their stories as having *both* agency *and* being determined by social norms and power-relations that required them to undergo FGC, describing having a lack of choice or feeling forced to undergo it.

In storying the FGC-event, participants made sense of and conceptualized their experience in light of dominant Western discourses and presumed hegemonic ideas about girls and women with FGC as victims of a violent event. Some talked from a victimized position, others talked back to such a position. Common to all participants was that they each engaged in varying kinds of contextualizing and talking back to assumed shared ideas about FGC in their new social setting. The examples are illustrative reminders that the storytelling context prompts participants to tell a certain kind of story in a certain way. Some stories seem not intelligible to tell, given the cultural context, but need to be rationalized and contextualized for participants to think of them as plausible to voice in the interview situation.

#### 4.2.1. Change talk: narrating from the position of a “now better-knowing” self

Condemnation through the eyes of the current context was another recurring theme permeating the material—and participants commonly engaged in various forms of “change talk”—accentuating how they now were thinking of FGC as a redundant event:

It should not continue, it should end, stop. As we have now gained more knowledge, the people have received more information, so this [FGC]—no one does it anymore, even in Somalia, it has been stopped. (woman in FGD)

Previous practices of FGC were made sense of in terms of being “backwards,” having a “traditional mindset,” and having a “lack of knowledge” as regards medical or religious aspects of the practices. Participants, young and older, positioned themselves against these imageries of the less known, by declaring how they or Somali people “now know better” and are opposed to FGC. Aalihya (18–23, unmarried) clarified:

Back then [in Somalia], you thought that [pharaonic] was required by religion, so we thought that what they say must be right—it [FGC] must be good. But then when we came here [to Sweden], and you became older, you have talked a lot about sex and stuff in class, and you have studied more religion and seen that it's not permitted.

From the position of a “now better-knowing” self, participants described themselves, parents, or the wider Somali community, as having acted according to what they thought was in the best interest of the child and/or in accordance with religious teachings. Rukhaya (24–28, unmarried) said:

Now when we know it's not allowed in the religion, we [sisters] say to our mom “look what you have done” … but she's not so knowledgeable about religion so that she can tell exactly what is right. … she's not *that* educated that she knew what was right and what was wrong, so that's why we can't say to her “you didn't do good.”

As Rukhaya drew on personal experiences, she also talked back to dominant Western narratives including the character of the evil villain or the uncivilized in a barbaric-civilized binary. Instead, the emphasis in her and others' accounts was on FGC as an act of care by loved ones. Drawing on discourses on what constitutes good parenthood and good religious practice, FGC was made sense of as rational given previous cultural scripts. Accordingly, participants staked out moral positions of being a good parent, having good intentions, or being a good Muslim acting in accordance with moral discourse. This could be interpreted as a way to accommodate both previous cultural scripts that required FGC, positioning it as desired and a social norm, and to the new position of opposition toward the practice and as something that is undesired and should end.

### 4.3. Stigma

Few participants spoke explicitly about stigma; however, the accounts showed how they navigated and related their experiences in different ways in relation to FGC being a stigmatized experience in Sweden. Few claimed to have taken part in FGC information through Swedish news media, TV, or newspapers; and only a few participants explicitly referred to “the media” as a source of stigmatizing messages about FGC. Instead, most related to an indirect, subtle, and internalized experience of stigmatization, given that FGC is not a social norm in Sweden.

Some shared experiences of feeling stigmatized in relation to the criminalization of FGC. In an FGD with older women, they continuously referred to an anecdote about a Somali family in their hometown. The story went, as told by a woman in her 50's.:

The daughter in primary school had told her teacher they would visit her family in Somalia during the summer. Suddenly the family was called in by the school nurse who had reported to the social authorities for fear of future genital mutilation. Social services told the mother they were not allowed to mutilate their daughter and they had to sign a paper to confirm they had received the information. “Not all Somalis want to circumcise their daughters, you know,” the women told me. “You feel suspected and haunted in a way, even though you're not approving of genital mutilation yourself.”

Sometimes, as in the above example, accounts of stigma were related to safeguarding measures; however, such examples were rare. More often, experiences of stigmatization in relation to FGC were related to social aspects. The two sisters Maryam (16–18 years, unmarried) and Bilan (20–23 years, unmarried) came from a home where their parents had decided not to have any of their four daughters undergo any type of FGC. They related in an FGD:

One time when I was in the store, a woman approached me... and she asked “why are you doing this [FGC]?” We said “it's a rule in our culture.” [She said:] “No, you must stop it, you must shout out to your country that this is no good” … and we didn't recognize the term [she used], but she showed us [cuts with her hands] so then we knew. [laughs]

The older women's and the young sisters' accounts illustrate how they managed ideas from outsiders about them being secret upholders or proponents of FGC.

Sometimes stigma was associated with the experience of having been exposed to FGC. When younger women talked about the stigma of carrying FGC, it was related to the prospects of getting married or having a partner. Yasmiin (16–18 years, unmarried) accounted for how she navigated what she assumed to be Swedish-Somali boys' expectations in relation to marriage:

When he asks if you are sunni or [pharaonic] and you're not sunni, he won't be happy … because this woman can't make you happy.

Yasmiin based her reasoning on discourses about pharaonic cutting and diminished sexual pleasure learned in Somalia. Among older women, stigma in relation to having been exposed to FGC was particularly salient in relation to gynecological examinations. Women recurrently told how they at some point had felt different and alienated by healthcare staff during gynecological examinations. Older women expressed how they prepared for healthcare providers' preconceptions about FGC or strong emotional reactions. Here is an excerpt from an FGD:

Luul: When they examine you, they cry.

Khadiija: Saying, ‘I'm sorry this happened to you.'

Luul: The nurses look [at you] and get all shocked.

Khadija: You can tell they get sad.

Luul: I'm afraid they will react like that.

Interviewer: Afraid of their reaction?

Khadija translates for Luul: Afraid that they will look, say stuff, the questions they ask, how much pain you feel.

Participants repeatedly stressed that they sensed that FGC stigma was associated with the Somali group. A scenario often-cited was how young women—themselves, friends, or daughters—had been targeted by school nurses when seeking help for menstrual pain. Batuulo (18–23, unmarried), who worked in a youth organization with anti-FGC advocacy and support in relation to FGC, related:

I particularly encounter young women who tell me that they are treated in a [certain] way in the Swedish healthcare system, like “ah, maybe you'll have trouble giving birth because you're mutilated.” They say that without knowing, without having examined me, or just because I'm from this place [Somalia]—then they call me mutilated, just because I have menstrual pain, they'll bring this up [genital mutilation]. … so it's very dramatic.

Many experienced that healthcare staff jumped to the conclusion that their symptoms were caused by FGC, regardless of whether having undergone any type of FGC or not. Most illustrative of this situation was probably the story of Filhan (16–18 years, unmarried). She explained:

I had appendectomy some years ago, but when I complain about still having aches, they sent me to the gynecologist to ensure it's not female circumcision. And I have already told [them]: I'm not [circumcised]! But they're like: “with these symptoms, it could be this [FGC]” … I have noticed among my friends from other countries, that they don't get these questions “are you genitally mutilated” … And [at the doctors] I'm like “no,” and they're like: “are you sure? Do you remember when you were a kid” and blablabla.

In the case of Filhan, her story was not deemed trustworthy by the healthcare providers she encountered, as they kept insisting that her symptoms fitted their picture of FGC. What the girls' and women's stories also show is how they navigated outsiders' ideas about FGC where FGC stigma was attached to the women's bodies and Somali appearance, regardless of actual FGC experience. Batuulo related a situation in school where the teacher had targeted her in a general discussion about FGC:

We had a teacher, and you could tell he wanted me to talk about it. “What do you think about this female genital mutilation?” I can see why he asked … but he singled me out … I told him it's not okay because I have noticed it only happens to Somalis, or Somali girls.

A common comment about this situation was that “we cannot hide” from peoples' anticipations, referring to Somalia as a high-prevalent country of FGC. This association was endorsed and sympathized with by some and resisted by others. The advantage, some argued, is that FGC becomes acknowledged as a potential concern affecting the Somali group. Others talked back to representations associating Swedish-Somalis with FGC, for instance, by trying to detach FGC from Somali ethnicity by engaging in change talk (see previous section) or stating that it is not exclusively a Somali phenomenon. Yet, others expressed feeling stigmatized and sterotypified by service providers and others when “the first thing she [the nurse] asks when she looks at me: ‘Are you genitally mutilated?” as stated by Filhan.

## 5. Concluding discussion

This study highlights how Swedish-Somali girls and women construct their stories about FGC in relation to dominant condemnatory discourses about “female genital mutilation” in their efforts to make sense of personal experiences. Personal experiences were narrated in relation to what participants assumed to be societal ideas about FGC, in particular in relation to notions of harm, agency/force, and victimization.

That participants had to actively navigate and negotiate dominant condemnatory discourses on FGC was visible in relation to the social context—in the interview situation with the researcher and other participants in FGDs—and in relation to stories about navigating outsiders' expectations. Participants engaged in various narrative strategies for *talking through* and *talking back* (hooks, 1989) to dominant condemnatory discourses. This became clear through the absence of clarification and abridged comments implying “we all know—no need to clarify” (talking through), or stressing in-group variation, offering contextualization, and engaging in the un-doing of stereotypes (talking back). These strategies can be understood as an example of how they storied their experiences in relation to a grander narrative that was supposedly shared and known—a “standard tale” (Leonard, 2000, 2020).

In relation to the wider society, participants navigated societal expectations of them being in favor of FGC. The experiences resonate with a public discourse in Sweden that insists on FGC being a widespread yet hidden activity. Such claims are common in Swedish news media, despite plenty of research suggesting that there is generally a fast abandonment of the practice after the migration (the situation has been described in Johnsdotter, 2019). Being afraid to raise authorities' suspicions in relation to the criminalization of FGC was an example of how the participants had to navigate societal expectations regarding their attitudes toward FGC. More commonly, however, participants had internalized stigma related to FGC. As reported in the previous literature (O'Neill and Pallitto, 2021), girls and women in the current study explained how they prepared for strong emotional reactions or preconceptions about FGC from healthcare staff—what Jacobson et al. (2022) identified as women conducting “emotional health work.” The present study shows that this work was conducted also outside the healthcare context, and regardless of actual experiences of genital cutting, i.e., also the young uncut participants had to negotiate an ascribed FGC label, for example, when seeking help for menstrual pain. This situation is also described in a previous article where Swedish healthcare staff explain how concerns about FGC were directed at Somali girls (Palm et al., 2021). The association made by others between Somalis and FGC was described as an attributed category that stuck to their bodies only by appearance. It was an association that invoked strong images and emotional responses in the other, what can be understood as a “sticky association,” to use Sara Ahmed's concept (Ahmed, 2014; p. 11, 98). The findings suggest that FGC as a sticky association with Somali ethnicity, risks having stigmatizing and stereotyping effects. As has been reported in previous studies among Somalis in Canada and the UK (Khaja et al., 2010; Karlsen et al., 2020; Parikh et al., 2020), the findings in the present study point to a tendency of overgeneralization where girls and women of Somali background are singled out, misjudged, or are having their situation reduced to a matter of alleged FGC. Ultimately, such an approach has been shown to lead to suboptimal healthcare and diminished trust in service providers and prevention efforts (Essén et al., 2002a; Khaja et al., 2010; Karlsen et al., 2020). This adds to poorer health outcomes and barriers to accessing proper care and support as already faced by Somali women in Sweden and in other diaspora settings (Essén et al., 2002b; Esscher et al., 2014; Karlsen et al., 2020).

Previous literature on FGC has shown the impact of migration on the reformulation of previous experiences of FGC (Catania et al., 2007; O'Neill and Pallitto, 2021). The present study further displays how both condemnatory discourses in Sweden *and* Somalia, on national, community, and relational levels, have informed how participants make sense of FGC. Confirming previous studies, the Swedish-Somali girls and women in this study had internalized an ideology of FGC as bad, wrong, and undesired (c.f. Gele et al., 2012; Wahlberg et al., 2017; Johansen, 2022). They were affected by discourses framing FGC as harmful to health and sexuality, as well as un-Islamic (Wahlberg et al., 2019; c.f. Johansen, 2022). “FGC” was in most cases synonymous with infibulation (Powell and Yussuf, 2018; c.f. Johansen, 2022), at least in relation to health aspects. Participants explained how they had developed negative attitudes toward FGC as a result of religious convictions or experiences of harm—their own experiences or those heard of from significant others. Notions of harm were also based on hearsay and anecdotes within the Somali community in Sweden and Somalia. Circulating stories about the “sexually ‘dead”' infibulated woman was an example of this. As has been described in previous research, negative attitudes toward FGC were also informed by dominant condemnatory discourse in the Swedish migration context, which emphasizes FGC as a violent and harmful practice (Gele et al., 2015; Wahlberg et al., 2019; Ziyada et al., 2020). This picture of FGC as related to by the participants concurrently resonates with discourse in Somalia, where FGC is increasingly condemned on a similar basis, partly due to decades of global efforts to end FGC (Powell et al., 2020). Based on these sources, an image of the “cut woman” was constructed. In terms of harm, personal experiences of FGC were positioned against and made sense of in relation to this imagined other (infibulated) woman, associated with victimization, lifelong suffering, and sexual problems. While participants were affected by anti-FGC discourse, especially the emphasis on the negative health and sexual effects, the construction of a binary between sunna and pharaonic cutting, where the stigma of anti-FGC discourse was related to pharaonic cutting, seemed to offer discursive space for identity construction and position that accommodated to both the condemnatory setting in migration, discursive changes on FGC in Somalia, and personal experiences and beliefs. Similar conclusions are presented in a recent study among Sudanese and Somalis in Norway, but rather among the older generation (Johansen, 2022). The way participants related notions of long-term harm to infibulation while generally excluding clitoridectomy could be interpreted as their statements being more influenced by the discursive change from infibulation to sunna cutting in Somalia, than of Western dominant discourses on harm which include all types of FGC. Another plausible explanation could be that anti-FGC messages in Sweden tend to emphasize the health effects of infibulation without discriminating between specific types of cutting (e.g., Östergötland County Administrative Board, 2015). This could have the effect that all other kinds of procedures are detached from the FGC category.

In terms of prevention efforts and treatment, the participants' conceptualization of sunna versus pharaonic cutting has at least two implications. First, young women with sunna cutting positioned themselves through a negative definition of pharaonic cutting—as something they were not. This might be a consequence of the stigma related to FGC, and signals that no alternative (positive) identification seems available that can combine having undergone FGC with not necessarily feeling harmed. The exception was the young woman, Yasmiin. Drawing on discourses about health, religion, and modernity, the separation of pharaonic and sunna cutting seemed, for her, to provide a progressive, modern, and “whole” self-identity. On the contrary, for women with infibulation, there seems to be a repertoire of language and labels for self-categorization within the survivor or victim paradigm. Yet, the “mutilation” discourse—as implied by the very term mutilation—offers few other (positive) options for self-categorization other than maimed. Second, in efforts to end FGC, there has been a tendency to emphasize “worst case scenarios” (Obiora, 1997; p. 53) of possible health, mental, and sexual outcomes of the FGC event. These are often based on alleged consequences of infibulation, without differentiation between specific types of cutting and their potential related risks (Obiora, 1997; Gruenbaum, 2005). The present study further underscores what has previously been argued, namely the value of discerning between various kinds of cutting and their specific potential health risks. Otherwise, some girls or women might, as suggested by Earp and Johnsdotter (2021), expect “the worst” from their cutting, irrespective of their own experiences from FGC, or, as suggested by the present study, some may discard FGC messages altogether, seeing them as unrelated to their own experiences.

So what kind of story is this? In social science research on storytelling, it has been recognized that publicly circulating stories have a great impact on social life (e.g., Plummer, 1995; Loseke, 2011). In his seminal book, *Telling Sexual Stories*, sociologist Ken Plummer (1995) illustrates how what he called “sexual stories,” e.g., “coming out stories” or “rape stories,” when becoming public, create space for others to draw upon when narrating their own experiences. In this way, it can be argued that stories may become interactive. At an individual level, people use stories to organize, understand, and interpret their experiences. Scholarship on FGC has increasingly cautioned that a drawback from an anti-FGC discourse that categorizes cut girls and women as “mutilated” may negatively impact their body image, self-esteem, and sexual self-image, insofar as girls and women start defining themselves in terms of their “mutilation” (Johnsdotter and Essén, 2016; Earp, 2021). There is now a myriad of studies supporting this notion (Johnsdotter and Essén, 2004; Ahmadu, 2007; Malmström, 2016; Villani, 2017; Ziyada et al., 2020), reporting how some women have incorporated the standard narrative of themselves as “mutilated” (Villani, 2017; Ziyada et al., 2020), disfigured (Jordal et al., 2022), or victimized (Vloeberghs et al., 2011). Similarly, a recent systematic review of qualitative research on psycho-social wellbeing after FGC synthesizes how some women with FGC report feelings of shame, stigma, inferiority, or of being different in migration settings (O'Neill and Pallitto, 2021). In contrast to these findings, the present study found that such a narrative did not seem to provide a meaningful language or a sufficient frame to make sense of one's own experiences. In contrast to the standardized story about FGC, this study indicates that there is no *one* uniform story among the interviewees. The study found that the Somali girls and women had internalized an ideology of FGC as bad, wrong, harmful, and undesired, yet, on a personal level, navigated experiences of not necessarily themselves feeling harmed, wronged, or violated. Simultaneously, participants negotiated previous cultural scripts that constructed FGC as religious, morally good, and desired. Participants used various narrative strategies such as contextualization and engaging in change talk, or positioning against an imagined cut woman to make sense of these experiences in the new Swedish context. This can be seen as an example of how stories are created, negotiated, and exchanged in relation to the social context and the receiver of the story (Plummer, 1995). Where previous studies have contributed to an understanding of how new labels, categorizations, and possible social identities such as “the mutilated”—offered through prevention strategies (e.g, campaigns and awareness raising efforts)—may affect girls and women already exposed to FGC, the present study furthers the understanding of girls' and women's agency in constructing and telling stories about FGC in a migration setting that condemns FGC. Overall, the study showed that participants did not carry a single “grand narrative” about FGC, but still had to navigate and negotiate such a narrative. Common to most was that they engaged in various kinds of counter-storying in their efforts to make sense of their unique experience. Navigation was conducted both at an intrapersonal level through continuous identity work, and also in relation to the social context in interpersonal encounters, i.e., with service providers and others, among which the standard tale has become a truth.

Some discourses and stories, in a given time and place, attain more cultural currency than others (Foucault, 1981; Plummer, 1995). In Sweden, “the standard tale” of FGC is an official story about FGC that has gained greater cultural currency than others. Overall, the study shows how participants, when trying to make sense of their own experiences, had to negotiate a dominant story about FGC including ascribed identities, experiences, labels, and terms that were not necessarily developed from their own realities. One possible implication of the dominance of the standard tale is that stories that counter the listener's expectations are silenced or not deemed trustworthy, such as in the example of Filhan. If a stereotyped story, like the “standard tale,” informs the professional encounter rather than the personal experience, it might have stigmatizing and silencing effects and may distort communication. Brian Earp, the bioethicist and notorious defender of all children's right to avoid non-consensual genital modifications, sympathetically suggests that rather than forcing victim status on an individual, “an alternative approach would be to acknowledge the diversity of outcomes, meanings, and interpretations surrounding distinctive types of genital cutting across societies, both positive and negative, and allow affected individuals to decide for themselves whether they wish to be treated or seen as victims of ‘mutilation”' (2021; p. 1880).

## Data availability statement

The datasets presented in this article are not readily available because all relevant data is presented in the article. Requests to access the datasets should be directed to camilla.palm@mau.se.

## Ethics statement

The studies involving human participants were reviewed and approved by the Swedish Ethics Board (Dnr 2014/620; Dnr 2020-00724). Written informed consent for participation was not required from the participants in accordance with the national legislation and the institutional requirements. Participants gave verbal informed consent at the beginning of their interviews, which was documented by audio recording.

## Author contributions

BE funded, administered, designed the project, and edited and revised the manuscript for intellectual content. EE and CH supervised the manuscript, contributed to the conceptualization of the analysis, and edited and revised the manuscript for intellectual content. CP designed the research, conducted and analyzed the qualitative data, and wrote original draft and edited and reviewed the manuscript. All authors have read and approved the final manuscript.

## Footnotes

^1^“Så ska vi stoppa könsstympningarna” [This is how we will stop the genital mutilations], debate post [debattinlägg] in the Swedish newspaper *Expressen*, by Maria Arnholm, Minister of Gender Equality at that time, about the Governments work on gender equality, in *Expressen* 8 September 2013.
